# Effects of N-acetylcysteine on systemic lupus erythematosus disease activity and its associated complications: a randomized double-blind clinical trial study

**DOI:** 10.1186/s13063-023-07083-9

**Published:** 2023-02-21

**Authors:** Mitra Abbasifard, Hossein Khorramdelazad, Abdolrahman Rostamian, Mohsen Rezaian, Pooya Saeed Askari, Gholamhosein Taghipur Khajeh Sharifi, Moein Kardoust Parizi, Mobina Taghipour Khajeh Sharifi, Seyed Reza Najafizadeh

**Affiliations:** 1 Non-Communicable Diseases Research Center, Rafsanjan University of Medical Sciences, Rafsanjan, Iran; 2 Department of Internal Medicine, Ali-Ibn Abi-Talib Hospital, School of Medicine, Rafsanjan University of Medical Sciences, Rafsanjan, Iran; 3 Molecular Medicine Research Center, Research Institute of Basic Medical Sciences, Rafsanjan University of Medical Sciences, Rafsanjan, Iran; 4 Rheumatology Research Center, Vali-Asr Hospital, Tehran University of Medical Sciences, Tehran, Iran; 5 Biostatistics Group, Division of Epidemiology & Health Sciences, The University of Manchester, Manchester, UK; 6 Department of Surgery, Ali-Ibn-Abi-Talib Hospital, Rafsanjan University of Medical Sciences, Rafsanjan, Iran; 7 School of Dentistry, Shiraz University of Medical Sciences, Shiraz, Iran

**Keywords:** Systemic lupus erythematosus, N-acetylcysteine, Anti-oxidant, BILAG, SLEDAI

## Abstract

**Backgrounds:**

N-acetylcysteine (NAC) has broadly been used as an anti-oxidant agent in various types of diseases. This study aimed to assess the effect of NAC on the systemic lupus erythematosus (SLE) disease activity and outcome.

**Methods:**

In this randomized, double-blind clinical trial study, 80 SLE patients were recruited that were classified into two groups: 40 patients received NAC (1800 mg/day; 3 times per day with 8-h intervals) for 3 months and 40 patients as the control group received normal therapies. Laboratory measurements and disease activity based on the British Isles Lupus Assessment Group (BILAG) and SLE Disease Activity Index (SLEDAI) were determined before the initiation of treatment and after the study time period.

**Results:**

A statistically significant decrease in BILAG (*P*= 0.023) and SLEDAI (*P*= 0.034) scores after receiving NAC for a 3-month period was observed. BILAG (*P*= 0.021) and SLEDAI (*P*= 0.030) scores were significantly lower in NAC-receiving patients compared to the control group after 3 months. The disease activity in each organ based on BILAG score after treatment indicated a significant decrease in the NAC group compared to the baseline level in general (*P*=0.018), mucocutaneous (*P*=0.003), neurological (*P*=0.015), musculoskeletal (*P*=0.048), cardiorespiratory (*P*=0.047), renal (*P*=0.025), and vascular (*P*=0.048) complications. Analysis indicated a significant increase in CH50 level in the NAC group after treatment compared to the baseline level (*P*=0.049). No adverse event was reported by the study subjects.

**Conclusions:**

It appears that the administration of 1800 mg/day NAC to SLE patients can decrease the SLE disease activity and its complications.

## Introduction

Systemic lupus erythematosus (SLE) is a chronic autoimmune inflammatory connective tissue disorder that affects multiple organs [1]. The production of pathogenic autoantibodies and irregular immune responses are involved in the pathogenesis of SLE which leads to clinical and serological manifestations [2, 3]. The disease has a wide range of clinical manifestations, including rash, oral ulcer, arthralgia, and life-threatening involvement of internal organs, the most common of which is lupus nephritis [1]. Manifestations of SLE are very variable and intermittent with unpredictable recurrences, and exacerbations can characterize it during immune system attacks to various organs [4]. During SLE pathogenesis, organ damage might be occurred due to the precipitation of antibodies and immune complexes on vasculature structures [5].

The 5-year survival rate of SLE patients has increased to 90% in most treatment centers due to early diagnosis and improvement of therapeutic factors to control the disease and relevant complications [6]. Therefore, a remarkable enhancement in the survival rate has led to an increased quality of life of SLE patients. Immunosuppressive treatments, including corticosteroids, hydroxychloroquine, azathioprine, cyclophosphamide, and mycophenolate mofetil, have been used in the treatment of patients with moderate to severe lupus. However, many of these patients encountered treatment failure, and some experienced relapses during treatment with preservative dosage [7]. Evidence showed that mitochondrial dysfunction in patients with lupus leads to increased reactive oxygen metabolites (ROMs) [8]. This phenomenon leads to an ATP drainage, a reduction in glutathione, and a decrease in necrotic death-predisposing cells, among the leading causes of inflammation [9]. To overcome the production of these metabolites, the administration of N-acetylcysteine (NAC) as an anti-oxidant factor is one of the new therapeutic strategies in SLE patients. NAC is an anti-oxidant drug that improves the disease’s activity by blocking the mammalian target of the rapamycin (mTOR) signaling pathway in T cells [10]. An animal study also found that administering aminothiol compounds like NAC and cysteamine (CYST) improved the disease’s outcome in mouse models of lupus [11].

As an adjuvant, NAC therapy is a controversial issue among rheumatologists in managing and controlling lupus disease. Therefore, considering the high prevalence of lupus in Iran, the importance of using anti-oxidant agents in controlling lupus activity, and existing contradictory on NAC therapeutic effects in patients with lupus, the present study aimed to explore the effect of NAC on decreasing SLE disease activity and its complications and outcomes.

## Study subjects and methods

### Participants

The present randomized, double-blind clinical trial study was performed among patients with SLE referred to the rheumatology clinic of Imam Khomeini Hospital, Tehran, Iran. SLE diagnosis occurred based on the American College of Rheumatology (ACR) revised criteria [12]. The inclusion criteria were ages more than 16 years old, patients with lupus, history of taking prednisone, azathioprine, and mycophenolate mofetil. Exclusion criteria also were pregnant or lactating patients or subjects with chronic infections, last month infections, bronchiectasis, severe and recurrent infections, smoking, patients with excessive use of anti-oxidants (daily and without a prescription) and acetaminophen, acute SLE flares threatening vital organs, and patients in need of treatment with intravenous cyclophosphamide treatment or those receiving biological drugs like rituximab and abatacept.

The approval of this study was granted by the ethical committee of the Rafsanjan University of Medical Sciences (IR.RUMS.REC.1397.100) and registered as a clinical trial in the Iranian Registry of Clinical Trials (IRCT20181030041500N2). In addition, informed consent was obtained from all cases or their families.

### Study design and procedures

All patients were routinely examined and periodically tested at baseline. These tests included cell blood count, kidney and renal function, urine analysis, and laboratory tests like anti-dsDNA, anti-nuclear antibody (ANA) titer, serum complement C3 and C4 concentrations, total hemolytic activity (CH50), and proteinuria, performed in the reference laboratory of Imam Khomeini Hospital. Then, the patients were randomly divided into 40 cases receiving NAC and 40 control subjects. The case group received NAC (manufactured by Osveh Pharmaceutical Company, 600-mg effervescent tablets) at a dose of 1800 mg daily three times per day in an 8-h interval basis for 3 months. The control group continued to use the same standard lupus medication regimen. The activity of lupus disease before and after treatment was measured according to the British Isles Lupus Assessment Group (BILAG) scoring system that evaluates neurological, musculoskeletal, renal, mucocutaneous, general, cardiorespiratory, vascular, and hematological manifestations (indicating score 0 as non-involvement and score 4 as the highest involvement rate) (47) as well as based on SLE Disease Activity Index (SLEDAI) score (48). SLEDAI index determines the risk of seizures, psychosis, organic brain syndrome, visual impairment, cranial neuropathy, lupus headache, vasculitis, cerebrovascular events (CVA) score 8, arthritis, myositis, urinary casts, hematuria, proteinuria, pyuria score 4, new rash, alopecia, mucosal ulceration, pleurisy, pericarditis, hypocomplementaemia, increased DNA binding score two and fever, thrombocytopenia, and leukopenia with score 1. All patients were told to avoid any change in the drug pattern and other nutritional or behavioral habits. It should be noted that a daily allowance dose of multivitamins containing 500 mg of vitamin C and 30 international unit (IU) of vitamin E was performed in all patients. Demographic data, laboratory findings, BILAG score, and SLEDAI score were recorded in pre-designed forms.

### Statistical analysis

All encoded data were inserted into SPSS version 25 software (IBM SPSS Inc., Armonk, NY, USA). For data analysis, the mean quantitative variables such as age, BILAG score, and SLEDAI score and the frequency of qualitative data such as sex were calculated. The Kolmogorov-Smirnov test investigated the normal distribution of scale data. Comparison of quantitative variables between the two groups was performed by independent *t*-test, paired *t*-test, and qualitative variables by Chi-square test. The effect size of the treatment was measured by calculating Cohen’s *d*. Data were expressed by either frequency (%) or mean ± standard deviation (SD) and comparisons with a *P*<0.05 was considered statistically significant.

## Results

### Characteristics of study subjects

In this study, we enrolled 40 patients with SLE (50%) receiving NAC and 40 SLE patients (50%) in the control group. Demographic data were compared between the two groups in Table 1, which showed no statistically significant difference. There were no significant differences between the two groups considering used drugs, blood indexes analysis, baseline lupus disease activity, and lupus disease activity based on each organ's type of involvement (Tables 1 and 2).

**Table 1:** Comparison of demographic variables in the two study groups

Variables		N-acetylcysteine	Control	***P*** value
Age (year)		36.1±0.1	37.0±5.5	0.899
Gender; male/female		15 (37.5%)/25 (62.5%)	8 (20%)/32 (80%)	0.370
SLE disease duration (year)		11.0±4.9	9.0±5.4	0.598
Hemoglobin (mg/dl)		12.1±0.8	12.2±1.6	0.428
Hematocrit (mg/dl)		35.7±3.0	36.0±0.4	0.435
White blood cell count (cell/m^3^)		5786±2159	6813±3658	0.369
Platelet (cell/m^3^)		248,065±91,202	314,600±81,624	0.024
ESR (mm)		35±20.9	33.2±25.1	0.871
AST (IU/L)		36.6±1.2	47.5±26.5	0.357
ALT (IU/L)		86.7±35.4	38.1±19.0	0.042
Anti-dsDNA (IU/mL)		71.47±11.92	63.32±12.76	0.342
ANA titer (IU/mL)		92.15±16.55	93.34±15.29	0.999
C3 mg/dL		45.70±6.94	43.8±721	0.999
C4 mg/dL		54.49±5.88	50.27±6.2	0.999
CH50 %		80.2±14.25	78±11.6	0.889
Proteinuria ≥ 0.5 g/24-h urine		21.48±2.45	19.65±4.12	0.671
BILAG score		6.65±1.73	6.52±1.52	0.672
SLEDAI score		2.65±0.70	2.71±0.72	0.435
Drugs	Prednisolone	36	34	0.599
	Hydroxychloroquine	26	34	0.283
	Azathioprine	14	14	0.627
	Mycophenolate mofetil + prednisolone+ hydroxychloroquine	8	8	0.655
	Prednisolone+ hydroxychloroquine	33	30	0.895
	Prednisolone+ azathioprine	14	10	0.999
	Prednisolone + azathioprine+ hydroxychloroquine	8	6	0.999

*SLE* systemic lupus erythematosus, *ESR* erythrocyte sedimentation rate, *AST* aspartate aminotransferase, *ALT* alanine aminotransferase, *dsDNA* double-stranded DNA, *IU* international unit, *C3* C3 component of complement proteins, *C4* C4 component of complement proteins, *CH50* 50% complement haemolytic activity, *BILAG* British isles lupus assessment group, *SLEDAI* Systemic Lupus Erythematosus Disease Activity Index

**Table 2:** Comparison of lupus disease activity based on the type of involvement of each organ in study groups at baseline level

Symptoms	N-acetylcysteine	Control	***P*** value
General	5.8±0.7	6.0±1.5	0.521
Mucocutaneous	35.0±6.1	33.8±2.1	0.34
Neurological	10±0.5	12.4±1.8	0.152
Musculoskeletal	8.3±1.1	8.1±0.2	0.891
Cardiorespiratory	3.1±0.9	3.2±0.1	0.627
Renal	2.3±0.2	3.0±0.5	0.256
Vascular	6.7±0.4	7.6±0.9	0.438
Hematological	8.0±0.1	7.1±0.2	0.892

### Effect of NAC therapy on SLE disease activity

**Fig. 1:**
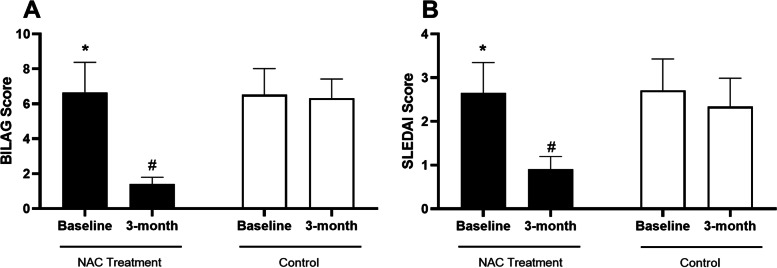
Bar graphs illustrates the lupus disease activity before and after treatment in each study group (NAC and control) based on BILAG (**A**) and SLEDAI (**B**) scores. The asterisk shows *P*<0.05 in the before-after comparison and # shows *P*<0.05 in the treatment-control comparison

Comparison of lupus disease activity based on BILAG and SLEDAI scores before and after treatment is presented in Fig. 1. Analysis indicated a statistically significant decrease in BILAG score after receiving NAC for a 3-month period (*P*= 0.023). The BILAG score was significantly lower in NAC-receiving patients compared to the control group after 3 months (*P*= 0.021). Nonetheless, there was no statistically significant alteration in the BILAG score in the control group at the 3-month timepoint compared to the baseline level (*P*>0.05, Fig. 1 A). In addition, after 3 months, the SLEDAI score was significantly lower in NAC-receiving patients compared to the baseline level (*P*= 0.034). After 3 months, the SLEDAI score was lower in the NAC-receiving SLE patients compared to the control group (*P*= 0.030). However, the analysis did not show a statistically significant alteration in NAC receiving group compared to the control group after 3 months (*P*>0.05, Fig. 1 B).

### Effect of NAC therapy on the disease activity in different organs

Our findings showed that disease activity in each organ based on BILAG score after treatment indicated a significant decrease in the NAC group compared to the baseline level in general (*P*=0.018), mucocutaneous (*P*=0.003), neurological (*P*= 0.015), musculoskeletal (*P*= 0.048), cardiorespiratory (*P*= 0.047), renal (*P*=0.025), and vascular (*P*= 0.048) complications, other than hematological score (Table 3).

**Table 3:** Comparison of lupus disease activity in each of the organs based on BILAG score before and after treatment in each of the groups

Symptoms	N-acetylcysteine			Control			Cohen’s ***d***
	Before treatment	After treatment	***P*** value	Before treatment	After treatment	***P*** value	
General	5.8±0.7	1.8±0.1	0.018	6.0±1.5	5.68±1.51	0.57	3.62
Mucocutaneous	35.0±6.1	2.61±0.1	0.003	33.8±2.1	31.9±1.8	0.67	22.97
Neurological	10±0.5	1.9±0.1	0.015	12.4±1.8	10.4±1.0	0.127	11.96
Musculoskeletal	8.3±1.1	3.3±1.0	0.048	8.1±0.2	8.0±1.9	0.366	3.09
Cardiorespiratory	3.1±0.9	2.1±0.9	0.047	3.2±0.1	3.6±1.1	0.752	1.49
Renal	2.3±0.2	0.3±0.01	0.025	3.0±0.5	2.9±1.3	0.343	2.82
Vascular	6.7±0.4	2.6±1.1	0.048	7.6±0.9	6.6±0.4	0.625	4.83
Hematological	8.0±0.1	5.9±2.8	0.677	7.1±0.2	7.1±0.2	0.237	0.61

### Effects of NAC therapy on the SLE complications

Blood and urine indexes, including anti-dsDNA, ANA, C3, C4, CH50, and proteinuria before and after treatment in each NAC-receiving and control group are presented in Table 4. Analysis indicated a significant increase of CH50 level in the NAC group after treatment compared to the baseline level (*P*= 0.049), but not in the control group. Other measurements did not significantly differ after 3-month treatment with NAC compared to the baseline level. Furthermore, none of the indices had a statistically significant alteration in the control group at the 3-month timepoint compared to the baseline level (Table 4).

**Table 4:** Comparison of laboratory measurements before and after treatment

Symptoms	N-acetylcysteine			Control			Cohen’s ***d***
	Before treatment	After treatment	***P*** value	Before treatment	After treatment	***P*** value	
Anti dsDNA (IU/mL)	71.45±3.55	68.25±3.48	0.72	63.35±4.89	61.52±5.20	0.78	1.52
ANA titer (IU/mL)	92.14±12.48	91.41±11.39	0.99	93.14±6.88	91.34±7.14	0.96	0.007
C3 (mg/dL)	45.25±5.66	59.08±6.21	0.67	43.55±7.21	43.36±8.11	0.66	2.17
C4 (mg/dL)	54.32±4.99	59.11±5.07	0.88	50.45±6.23	48.65±6.41	0.54	1.81
CH50 (%)	80.65±10.50	94.51±11.21	0.04	78.39±7.98	82.45±9.2	0.9	1.17
Proteinuria ≥0.5 g/24-h urine	21.46±5.22	19.66±4.89	0.99	19.78±3.14	18.71±15.6	0.99	0.08

*dsDNA* double-stranded DNA, *IU* international unit, *C3* C3 component of complement proteins, *C4* C4 component of complement proteins, *CH50* 50% complement haemolytic activity

### Safety of NAC therapy in SLE patients

Upon receiving 1800 mg/day NAC for a period of 3 months, no adverse event was reported by the study subjects. Therefore, NAC therapy is safe in SLE patients.

## Discussion

In the present study, the lupus disease activity revealed a significant decrease 3 months after treatment with NAC. General, mucocutaneous, neurological, musculoskeletal, renal, and vascular symptoms showed a significant improvement in the NAC group. While the anti-dsDNA, ANA, C3, and C4 levels had no significant difference between the two groups after the treatment, CH50 had a significant increase in the NAC group. The findings were consistent with other studies [10, 11, 13–16]. Oxidative damage occurs through targeted molecules, and oxidative modulator cascade products are associated with disease activity, organ damage, and concurrent illnesses in lupus. The intracellular anti-oxidant system naturally provides protection against reactive oxygen intermediates, and a reduced glutathione-dependent in vivo anti-oxidant system is available, and the NAC is capable of boosting this system [17]. This drug is a therapeutic strategy for lupus whose function is superior to oxidative stress.

The oxidative stress contributes significantly to the development of cardiovascular disease [18], which, in addition to renal failure and infection, is one of the leading causes of mortality and morbidity in patients with lupus [6]. Studies revealed that anti-oxidant therapy improves cardiovascular outcomes in patients with end-stage renal disease [19]. Based on the available evidence, oxidative stress is increased in lupus, which leads to immunodeficiency, organ trauma, and fatal illnesses. Phagocytic cells, through oxidative stress, are involved in tissue and organ damage [20]. Cytotoxic drugs tend to eliminate autoreactive cells and induce oxidative stress and cell death through the immune system, and suppression of oxidative stress is necessary for these patients. Accordingly, anti-oxidant therapy plays an essential role in limiting the toxicity of immunosuppressant treatments [21].

Additionally, the NAC as an adjuvant and a precursor to glutathione can improve lupus disease's clinical outcomes [11]. A study in lupus-induced mice demonstrated that the NAC prevents glutathione reduction, decreases autoantibody production, and increases nephritis frequency, leading to improved survival rates [11]. Based on the reduced glutathione in lupus patients [9], findings of a study on 36 patients with lupus under treatment with NAC (3 months) showed that NAC could have positive effects, such as safety, well toleration, and positive immunological and therapeutic effects in the patients [10]. Although NAC had no therapeutic effect at a dose of 1.2 grams per day, at the doses of 2.4 and 4.8 g per day, the glutathione begins to decrease, and disease and fatigue improvement were effective and safe [22]. In other studies, NAC’s benefits in the treatment of lupus have been shown, which could be due to increase HDL cholesterol levels [23] and reduce the incidence rate of cardiovascular disorders and renal failure [19].

## Conclusion

Taken together, according to the findings of this study, the addition of 1800-mg NAC to the treatment regimen of patients with lupus can improve the outcomes of the disease and decrease the activity and complications of lupus disease. Therefore, it is recommended that rheumatologists consider NAC supplementation in the treatment of these patients. It should be noted that we were unable to check the statistical comparison by the diet of the subjects as a confounder factor, which might have an effect on the final outcome. Future studies are also recommended to determine the clinical significance of this study in a larger sample size, with different doses of the drug, considering the severity of lupus disease and follow-up within 1 year. Moreover, NAC adjuvant drug therapy’s effect should be investigated in reducing the dose of the treatment regimen of immunosuppressive drugs in these patients.

## Data Availability

All data that support the conclusions of this manuscript are included within the article.

## Abbreviations

NAC: N-acetylcysteine
SLE: Systemic lupus erythematosus
BILAG: British Isles Lupus Assessment Group
SLEDAI: SLE Disease Activity Index
ROMs: Oxygen metabolites
mTOR: Mammalian target of rapamycin
CYST: Cysteamine
ACR: American College of Rheumatology
ANA: Anti-nuclear antibody
IU: International unit
SD: Standard deviation
